# 

**Published:** 1852-05

**Authors:** 


VOL. I. NEW SERIES. No. 1.

### THE NORTH-WESTERN

### MEDICAL AND SURGICAL

### JOURNAL.

PUBLISHED MONTHLY,

AT TWO DOLLARS PER ANNUM.

EDITED BY

W. B. HERRICK, M.D.,

PROFESSOR OF ANATOMY AND PHYSIOLOGY IN RUSH MEDICAL COLLEGE; SURGEON TO THE U. S. MARINE HOSPITAL, AND ONE OF THE SURGEONS OF THE ILLINOIS GENERAL HOSPITAL, ETC.

ASSISTED BY

H. A. JOHNSON, M.D.

MAY, 1852.

CHICAGO:

BALLANTYNE & CO., PRINTERS, PUBLISHERS, & BOOKBINDERS,

NEW POST-OFFICE BUILDINGS, COR. OF CLARK AND RANDOLPH-STS.,

OPPOSITE THE SHERMAN HOUSE.

1852.

VOL. IX. WHOLE SERIES. No. 1.

